# Mature results of a randomized trial comparing two fractionation schedules of high dose rate endoluminal brachytherapy for the treatment of endobronchial tumors

**DOI:** 10.1186/1748-717X-8-8

**Published:** 2013-01-07

**Authors:** Olivier M Niemoeller, Barbara Pöllinger, Maximilian Niyazi, Stefanie Corradini, Farkhad Manapov, Claus Belka, Rudolf M Huber

**Affiliations:** 1 Department of Radiotherapy and Radiation Oncology, Ludwig-Maximilians University Munich, Marchioninistr. 15, 81377, Munich, Germany; 2 Division of Respiratory Medicine and Thoracic Oncology, Ludwig-Maximilians University Munich, Munich, Germany

**Keywords:** Endobronchial radiotherapy, High dose rate brachytherapy, Lung cancer, Re-irradiation

## Abstract

**Purpose:**

To determine the efficacy of high dose rate endobronchial brachytherapy (HDR-BT) for the treatment of centrally located lung tumors, two different fractionation schedules were compared regarding local tumor response, side effects and survival. Mature retrospective results with longer follow-up and more patients were analyzed. Initial results were published by Huber et al. in 1995.

**Methods and materials:**

142 patients with advanced, centrally located malignant tumors with preferential endoluminal growth were randomized to receive 4 fractions of 3.8 Gy (time interval: 1 week, n = 60, group I) or 2 fractions of 7.2 Gy (time interval: 3 weeks, n = 82, group II) endobronchial HDR-BT.

Age, gender, tumor stage, Karnofsky Performance Score and histology were equally distributed between both groups.

**Results:**

Local tumor response with 2 fractions of 7.2 Gy was significantly higher as compared to 4 fractions of 3.8 Gy (median 12 vs. 6 weeks; p ≤ 0.015). Median survival was similar in both groups (19 weeks in the 4 fractions group vs. 18 weeks in the 2 fractions group). Fatal hemoptysis was less frequent following irradiation with 2 × 7.2 Gy than with 4 × 3.8 Gy, although the difference did not achieve statistical significance (12.2% vs. 18.3%, respectively. p = 0,345). Patients presenting with squamous cell carcinoma were at higher risk of bleeding compared to other histology (21.9% vs. 9%, p = 0,035).

Multivariate analysis with regard to overall survival, revealed histology (p = 0.02), Karnofsky Performance Score (p < 0.0001) and response to therapy (p < 0.0001) as significant prognostic factors. For patients showing complete response the median survival was 57 weeks, while for patients with progressive disease median survival time was 8 weeks, p < 0.0001.

The KPS at the start of the treatment was significantly correlated with survival. Patients presenting with a KPS ≤ 60 at the start had a significantly (p = 0,032) shorter survival time (10 weeks) than patients with a KPS > 60 (29 weeks).

Moreover, the Karnofsky Performance Score of most patients improved during therapy (p = 0,001), suggesting successful palliation of cancer associated symptoms.

Multivariate analysis with regard to local tumor control found no significant factors.

**Conclusion:**

Endobronchial HDR-BT is an effective local treatment for advanced centrally located malignant tumors with endoluminal tumor growth. Local tumor response was significantly higher after HDR-BT with 2 × 7.2 Gy.

## Introduction

Lung cancer is the leading cause of cancer associated deaths in the United States and Europe [1,2] with the majority of patients presenting with metastatic disease [3]. Although substantial improvements in terms of survival time and quality of life have been achieved over the last decades [4,5], palliation of local and systemic cancer associated symptoms remains one main objective in the treatment of these patients. Local symptoms including dyspnea due to airway obstruction, cough and hemoptysis respond to chemotherapy [6] and external beam radiotherapy [7]. Nonetheless, in a substantial number of patients, local symptoms may not be treated sufficiently [7] with more than 50% of the patients still suffering from cough, dyspnea and hemoptysis [8] stressing the need for further therapeutic strategies.

In this context brachytherapy is highly effective in palliating local symptoms like bleeding, cough and airway obstruction, yielding improvements in the majority of patients [9-16].

Although randomized data demonstrated a more sustained palliative effect of external beam radiotherapy (EBRT) when compared to a single dose brachytherapy, this was achieved at the expense of more acute side effects in the EBRT group and a substantially longer treatment time (10 – 12 days for EBRT versus a single treatment session for brachytherapy) [17].

Thus, especially in patients with limited prognosis presenting with local symptoms, high dose rate brachytherapy (HDR-BT) offers a number of advantages including short treatment time, a relatively fast onset of treatment response and less acute side effects, probably by limiting irradiated volumes of normal tissue.

Brachytherapy has also been proven to be clinically useful in order to prolong the effect of laser recanalization and prevent stent obstruction by tumor overgrowth [18].

With curative intent, brachytherapy has been utilized with some success as a boost to conventional external beam radiotherapy [19,20] or as definitive treatment for stage I lung cancer [21]. However, in some series with longer follow up, relatively high rates of bronchial obstruction and fatal bleedings had been observed [19,21,22]. One non-randomized study compared different radiation doses (single dose of 20 Gy or 15 Gy at 1 cm from the central axis of the radiation source). In this study, multivariate analysis identified high single dose as a risk factor for fatal hemoptysis [22].

Although the effectiveness of HDR-BT in a palliative context is proven, no randomized data were available concerning the questions of optimal treatment dose and fractionation. Therefore we initiated a prospective randomized trial comparing two fractionation schedules in palliative lung cancer treatment. Interim results of this study obtained up to November 1993 were presented in 1995 [23]. Here we present retrospective a analysis with more patients and longer follow-up.

## Methods and materials

### Patients selection and randomization

After receiving informed consent, one hundred and forty two patients were included in the trial. Patients were eligible when the following criteria were met: Histologically or cytologically proven malignant lung tumor, tumor localization affecting the trachea, the main or lobar bronchi with a substantial occlusion of the lumen as determined by bronchoscopic examination, exclusion of other treatment options such as surgery, external beam radiotherapy, or chemotherapy, no concurrent tumor treatment. Randomization was done by a flipping coin procedure. There was no selection of the patients regarding age and gender, histological findings, tumor stage, or Karnofsky Performance Status (KPS). The study was approved by the local ethics committee and followed procedures were in accordance with the Helsinki declaration 1975 as revised in 1983.

### Brachytherapy procedure

Group I received a total dose of 15.2 Gy, delivered in 4 fractions of 3.8 Gy at 1 cm from the source axis at weekly intervals. Group II received a total dose of 14.4 Gy in two fractions of 7.2 Gy at 1 cm from the source axis with a time interval of three weeks. The dose concept was chosen in the early 1990s according to personal experience of therapists, which from a modern point of view seems somewhat old-fashioned. Brachytherapy was mainly carried out on an outpatient basis. Bronchoscopy was performed with the patient receiving topical anesthesia to determine the field intended for treatment. The treatment area was marked externally by fluoroscopy depending on the endoluminal tumor extension. Subsequently a guidewire was placed through the instrumentation channel of the endoscope. After removal of the bronchoscope, a shortened gastric tube with an external diameter of 0.5 mm was inserted via Seldinger technique over a guidewire and placed along side of the tumor region. The irradiation applicator was placed into the tube and taped to the tip of the nose to prevent dislocation. For brachytherapy a ^192^Ir HDR remote afterloading unit (Gammamed IIi, Isotopentechnik Dr. Sauerwein, Haan, Germany) was used.

Dose was calculated using the Plato TPS program. The treatment length was determined by bronchoscopy according to the endoluminal tumor extension including a safety margin of 10 mm. The dose was prescribed to 10 mm distance from the source axis.

### Baseline data

Prior to randomization, the following baseline tests were performed: Routine blood chemistry, lung function tests, fiberoptic bronchoscopy, plain chest radiographs and computed tomographic scans. All patients were staged according to the international staging system for lung cancer as recommended by the American Joint Committee on Lung Cancer in 1988. Histologic classification was done following the guidelines of the World Health Organization [24]. Karnofsky Performance Score was registered at the beginning and at the end of endoluminal irradiation.

Three months after brachytherapy a bronchoscopic control examination and a chest radiograph were performed to evaluate local control and tumor response. Local intraluminal tumor response as determined by bronchoscopy was defined as follows:

Tumor recurrence was characterized as intraluminal tumor disappearance followed by subsequent re-growth; tumor progression was defined as intraluminal persistence and further growth. No change was defined as persistent intraluminal tumor.

Complete remission was defined as no evidence of intraluminal tumor; partial response was defined as more than 25% tumor reduction. If complete remission, partial remission or no change was achieved, the response was rated as local control. The duration of local control was defined as time to progression judged by bronchoscopy. In case of a treatment failure during the follow up period an additional course of brachytherapy was allowed by the protocol.

Follow up was performed at regular intervals: The patients participated at the follow up study at defined intervals of three months. Fatal hemoptysis was defined as massive bleeding from the tracheo-bronchial tree leading to immediate death. A local intrathoracic problem was encountered when complications such as airway occlusion, pneumonia, or intrapulmonary metastasis were the main cause of death. Systemic complications were defined as distant metastasis causing death. Other causes were tumor unrelated disease or unknown reasons.

### End point

Survival time was considered the main endpoint. Assessment of local control as judged by bronchoscopy provided a second endpoint. The KPS was used to document the acute side-effect of brachytherapy or progression of disease.

## Statistics

All patients were analyzed on an intention-to-treat basis. Differences between both groups were considered significant with a p-value of less than 0.05. Survival plots were created using the procedure of Kaplan and Meier [25]. Testing differences in survival time was done with the log rank test. All statistical analysis was calculated with the Statistical Package for Social Science (SPSS/PC for Windows 10.0).

## Results

### Clinical data

142 patients were included in this study: 60 in group I (4 × 3.8 Gy) and 82 in group II (2 × 7.2 Gy). The imbalance in patient numbers results from a stop in randomization and consecutive patients were treated in group II. However, in retrospect it was not possible to identify the exact time-point at which the randomization was stopped. The baseline characteristics are shown in Table 1.

**Table 1:** Patient characteristics

	**Group I (n = 60)**	**Group II (n = 82)**
Median Age (range)	64 (39–86)	65 (40–88)
Male n = 49	(81.7%)	n = 53 (67.1%)
Female n = 11	(18.3%)	n = 27 (32.9%)
Tumor stage		
I	3.5%	2.3%
II	1.8%	0
IIIA	15.8%	15.2%
IIIB	26.3%	16.5%
IV	52.6%	65.8%
Histological features		
Squamous Cell Carcinoma	53.4%	40.2%
Adeno Carcinoma	17.2%	26.8%
Large-Cell Carcinoma	8.6%	4.9%
Oat Cell Carcinoma	15.5%	11.0%
Adenoid-cystic Carcinoma	0	2.4%
Other	5.1%	14.6%
Pre-Treatment: (more than 100% because of different treatment combinations)		
None	2.6%	12.9%
Surgery	29.4%	20.0%
Brachytherapy	2%	4.2%
Ext. Irradiation	41.2%	35.6%
Chemotherapy	23.7%	27.1%
ND-YAG laser	33.3%	38.8%
Stent	7.9%	9.9%
Combinations	45.2%	45.5%
Proximal localization of the irradiation probe		
Trachea	17.9%	20.8%
Right main bronchus	57.1%	55.8%
Left main bronchus	5.4%	7.8%
Right distal bronchus	12.5%	13.9%
Left distal bronchus	7.1%	2.6%

Group I consisted of 49 (81.7%) men and 11 (18.3%) women. Group II consisted of 53 (67.1%) men and 27 (32.9%) women. Median age was 64 years in group I and 65 years in group II. Most of the patients had advanced tumor stages. Only 21.1% in group I and 17.5% in group II had tumor stages below IIIB. In both groups more than 50% of the patients suffered from metastatic disease. The largest histologic subtype in both groups was squamous cell carcinoma (53.4% and 40.2%), followed by adeno carcinoma (17.2% and 26.8%).

Merely all patients were heavily pretreated. Most patients had various pre-treatments (97.4% in group I and 87.1% in group II), with a substantial number of patients having already received an external beam radiotherapy (41.2% in group I and 35.6% in group II) and a Nd-YAG laser therapy (33.3% in group I and 38.8% in group II). More than 45% in both groups underwent different treatment combinations.

A median KPS of 60 in both groups at study-begin illustrates the poor condition of the patients. After treatment the median KPS increased up to 70 (p = 0.005). This may indicate that brachytherapy causes little discomfort for the patients and delivers a good treatment option, especially in a palliative situation. Tumor localization was similar in both groups with a preference of the right bronchial system. The total irradiation dose was almost similar in both groups. 30% of the patients did not complete the intended treatment. Two patients (3.3%) in group I and 6 (7.3%) in group II received more than the initially intended dose due to tumor persistence.

### Local tumor response

The rates of local tumor response are shown in Table 2.

**Table 2:** Tumor Response in Group I and II at the time of the first control-bronchoscopy

	**Group I (4 × 3.8 Gy)**	**Group II (2 × 7.2 Gy)**
	**(n = 60)**	**(n = 82)**
Complete Response	4.1%	4.5%
Partial Response	44.9%	47.8%
No Change	8.2%	17.9%
Tumor Progression	42.9%	29.9%

**Table 3:** Causes of death

	**Group I (4 × 3.8 Gy)**	**Group II (2 × 7.2 Gy)**
Fatal hemoptysis	18.3%	12.2%
Other reason than tumor progression	13.3%	8.5%
Local problems	20%	32.9%
Unknown	15%	12.2%

**Figure 1:**
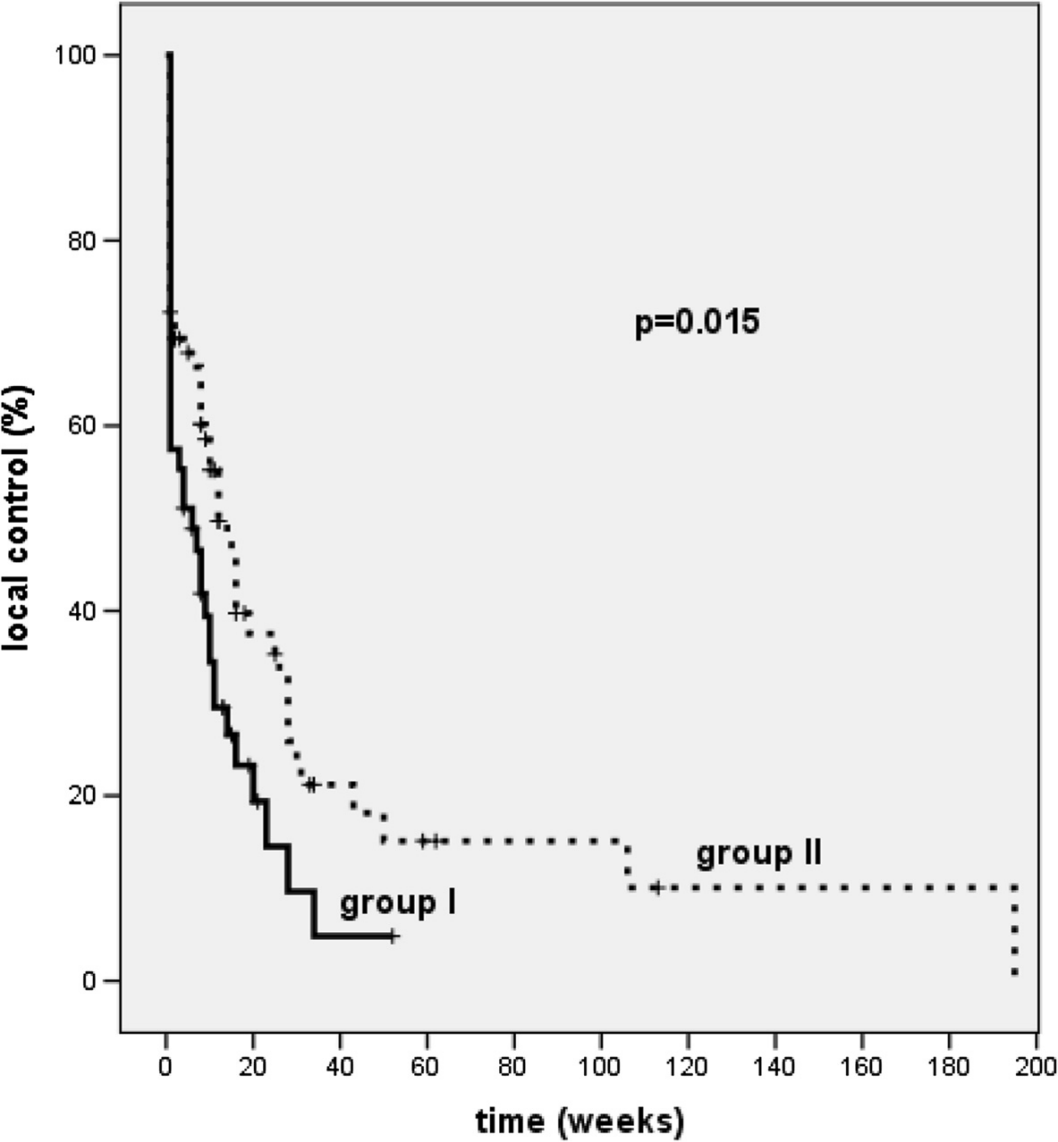
Kaplan-Meier curves for local control in patients treated with two fractions of 7.2 Gy (group II, broken line) or with 4 fractions of 3.8 Gy (group I, solid line) at 10 mm from the source axis (p = 0.015).

Local tumor control confirmed by bronchoscopy was significantly higher in group II compared to group I (p = 0.015). The mean time of local control in group I was 11 weeks whereas in group II it was 37 weeks (Figure 1). The duration of local control was defined as time from the last treatment to progression judged by bronchoscopy.

### Histological subtype

**Figure 2:**
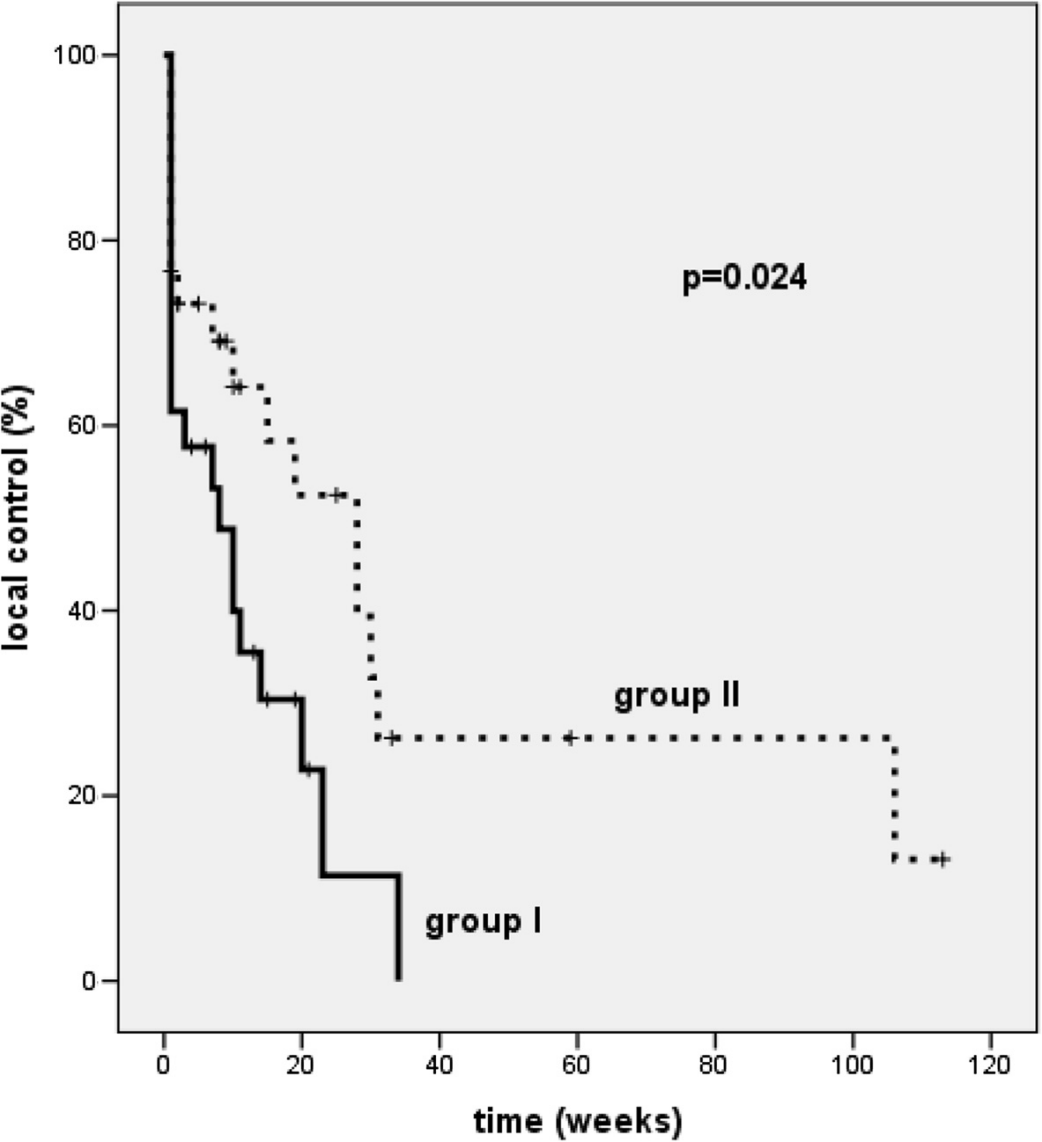
Kaplan-Meier curves for local control in patients with squamous cell carcinoma treated with two fractions of 7.2 Gy (group II, broken line) or with 4 fractions of 3.8 Gy (group I, solid line) at 10 mm from the source axis (p = 0.024).

Analysis of the histological subgroups revealed that patients with squamous cell carcinoma in group II (n = 30/82) had a statistically significant (p = 0.024) better local control (median local control 28 weeks versus 8 weeks) than in group I (n = 26/60) (Figure 2). However, the improvement in local tumor control in this subgroup did not translate into a statistically significant survival benefit (median survival 19 weeks for group II versus 14 weeks for group I, p = 0.469).

Patients with small cell lung cancer in group II (n = 9/82) also had a statistically significant (p = 0.028) better local control than the patients in group I (n = 6/60). The other histological subgroups did not show a significant difference in local tumor control comparing group I and II.

### Overall survival

**Figure 3:**
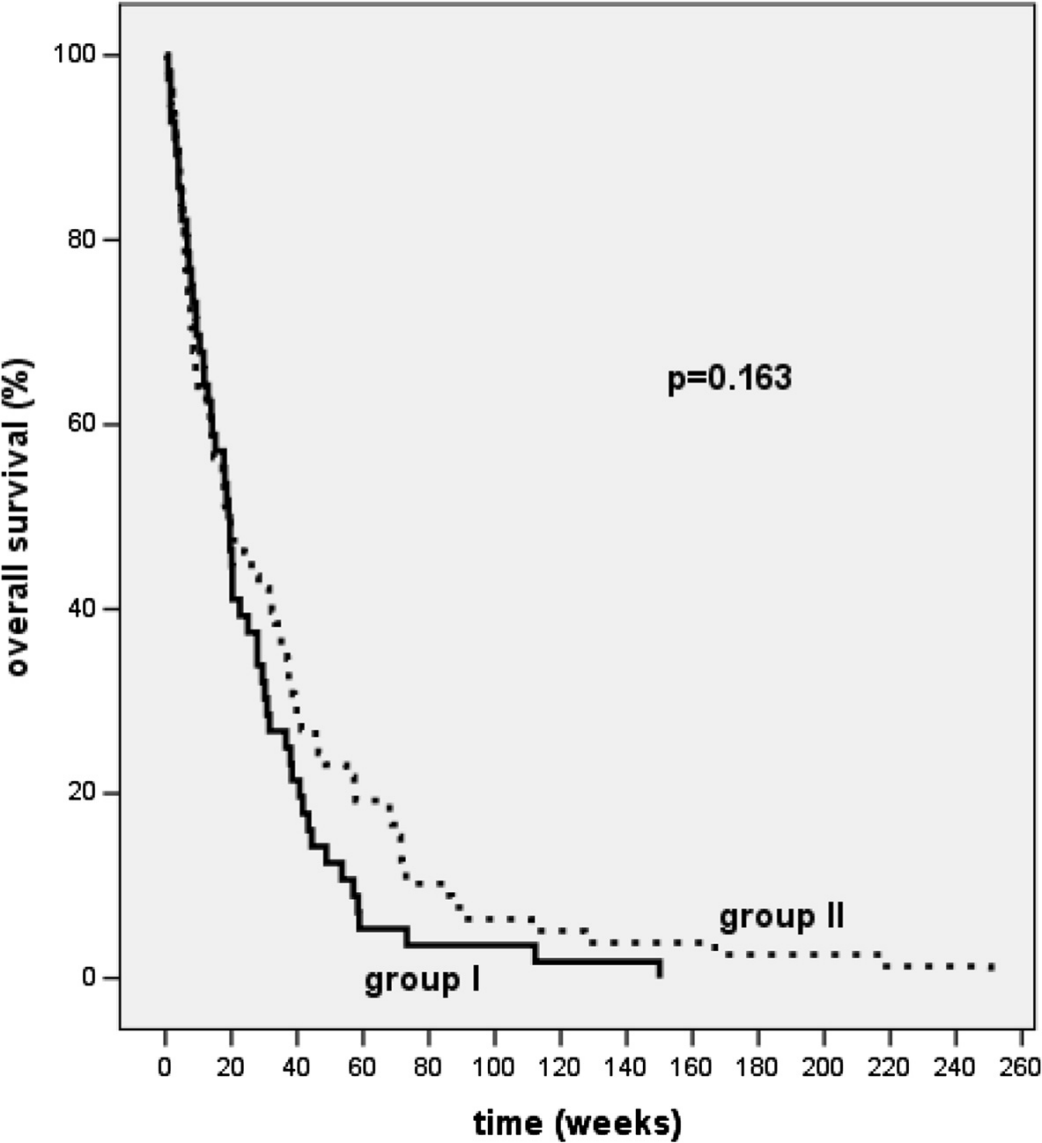
Kaplan-Meier curves for overall survival of patients treated with two fractions of 7.2 Gy (group II, broken line) or with 4 fractions of 3.8 Gy (group I, solid line) at 10 mm from the source axis (p = 0.163 n. s.).

One-year survival rates were 11.4% in group I and 21.1% in group II (median follow up of 20 weeks; range 1 to 251 weeks). Median survival in group I was 19 weeks, whereas in group II median survival was 18 weeks (p = n.s.) (Figure 3).

Adherence to treatment was similar in both groups. In group I 70 percent of the patients received the full dose, in group II 69.5 percent received the intended dose.

### Karnofsky performance score

**Figure 4:**
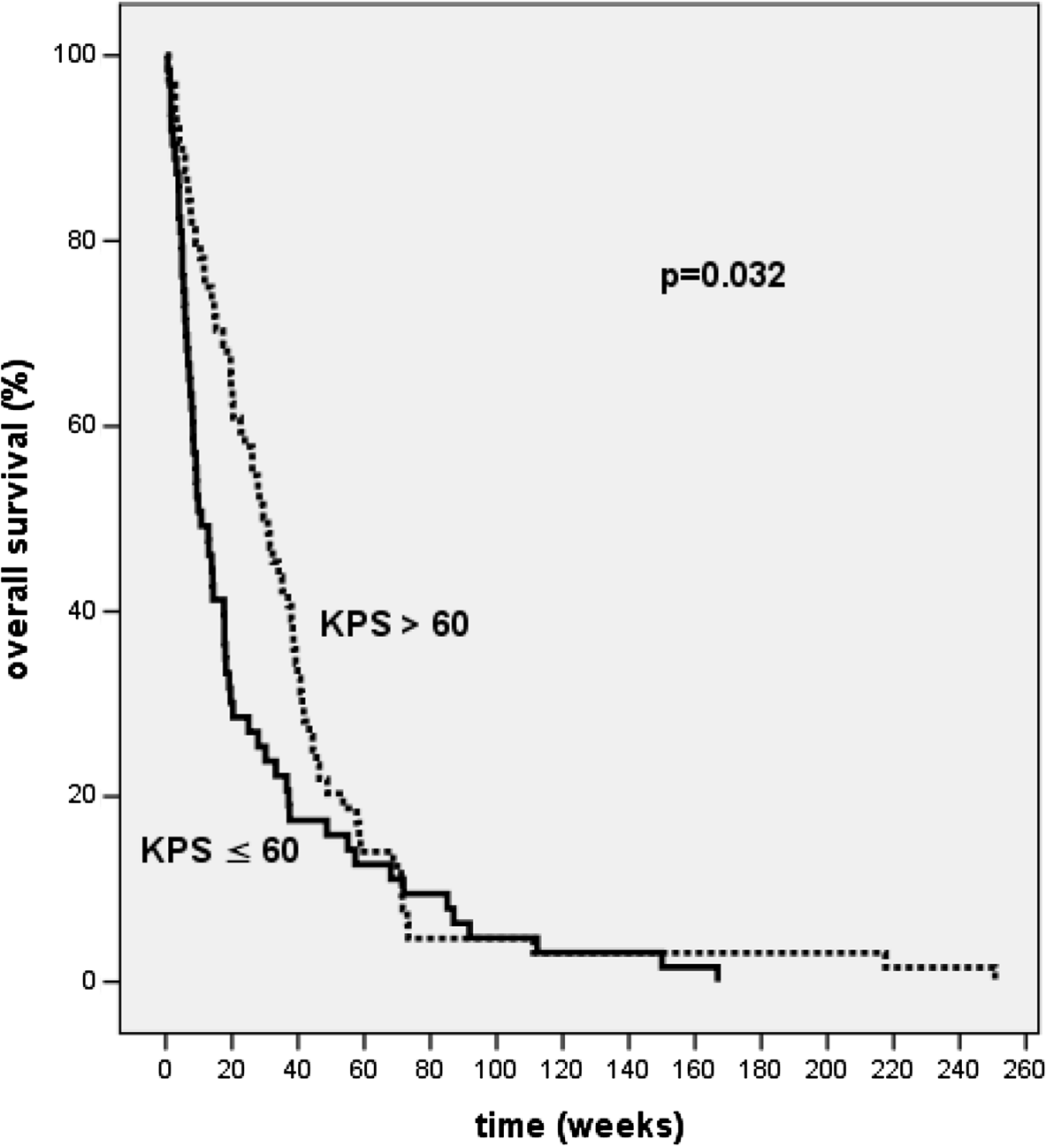
Kaplan-Meier curves for overall survival of patients presenting with a Karnofsky Performance Score of more (broken line) or less than (solid line) 60% (p = 0.032).

The KPS at the start of the treatment was significantly correlated with survival, with patients presenting with a KPS ≤ 60 at the start had a significantly (p = 0.03) shorter median survival time (10 weeks) than patients with a KPS > 60 (29 weeks) (Figure 4).

### The KPS of most patients improved significantly (p = 0.001) during the treatment. Causes of death

In both groups one third of the patients were dead after a follow up time of 12 weeks in group I and 9 weeks in group II. The underlying reasons were: Fatal hemoptysis which occurred in 11 patients (18.3%) in group I and in 10 patients in group II (12.2%). Patients with squamous cell carcinoma had a significantly higher risk of fatal hemoptysis compared to patients with other histological subtypes (p = 0,035).

8 patients in group I (13.3%) and 7 in group II (8.5%) died not related to tumor progression. Death in group I was caused by local problems in 12 patients (20%) and 27 patients in group II (32.9%). Causes of death were unknown in 9 patients in both groups respectively (15% vs. 12.2%) (Table 3).

## Discussion

Our data confirm former studies on high dose rate endobronchial brachytherapy (HDR-BT). We showed that endobronchial brachytherapy is an effective and safe therapeutic option in heavily pretreated patients with limited prognosis and low performance score. For patients with prior radiotherapy and limited prognosis HDR-BT offers a quick palliation of local symptoms, which is reflected in improved performance score.

Initially published results from the first 93 patients found no difference in either overall survival or local control with the two fractionation regimes [23]. The present analysis including 142 patients shows that better local control rates are achieved when using 2 fractions of 7.2 Gy compared to 4 fractions of 3.8 Gy. Furthermore, local remission time and consequently symptom relief was significantly better using 2 fractions of 7.2 Gy. One explanation for this phenomenon might be a quicker development of clinical effects due to the application of a higher irradiation dose per fraction. In addition, this regime is less invasive and probably more cost-effective - although this subject was not within the scope of the study. However, in the context of an advanced systemic disease, the improved rates of local control following irradiation with 2 fractions of 7.2 Gy did not translate into a survival benefit.

Concerning the weaknesses of this study, it must be stated that a weakness of this analysis is the fact, that in retrospect it was not clear when the randomization was stopped, which might have biased the results. Nevertheless, since the mature results are in accord with the initial results the authors believe that the results are valid.

Moreover, since randomization was done by a flipping coin procedure and no selection by known risk factors was preformed, the results might be biased.

Concerning the limitations of endoluminal brachytherapy, one must consider that although this technique provides an important option for the treatment of tumor-derived airway occlusions, the effects of brachytherapy are delayed in time so that HDR-BT is not suitable when immediate symptom elimination is necessary.

The data presented here confirm that the KPS is an important prognostic factor. Patients with a KPS < 60 had a significant shorter survival time. Moreover, the KPS improved after treatment. This might indicate an effective palliation of local symptoms and a lack of severe acute side effects.

In summary, HDR-BT provides an excellent treatment option for patients suffering from tumor-associated airway obstruction. Using two fractions with 7.2 Gy at 10 mm from the source axis provides superior local control in a randomized comparison with four fractions of 3.8 Gy. Thus, this regimen should be used for palliative radiotherapy in patients with limited prognosis presenting with local symptoms.

Supported by a grant of the Wilhelm-Sander-Foundation No.: 87.015.1 and 87.015.2.

## Competing interests

The authors declare that they have no competing interests.

## Authors’ contributions

OMN wrote the manuscript, BP acquired the data, MN did the statistics, SC did the statistics and provided the figures, FM contributed in data acquisition, CB developed the Concept, RMH did the acquisition of data and developed the concept. All authors read and approved the final manuscript.
